# Mesenchymal Stem Cells and COVID-19: Cure, Prevention, and Vaccination

**DOI:** 10.1155/2021/6666370

**Published:** 2021-05-06

**Authors:** Mehrdad Afarid, Fatemeh Sanie-Jahromi

**Affiliations:** Poostchi Ophthalmology Research Center, Department of Ophthalmology, School of Medicine, Shiraz University of Medical Sciences, Shiraz, Iran

## Abstract

COVID-19 disease has been a global health problem since late 2019. There are many concerns about the rapid spread of this disease, and yet, there is no approved treatment for COVID-19. Several biological interventions have been under study recently to investigate efficient treatment for this viral disease. Besides, many efforts have been made to find a safe way to prevent and vaccinate people against COVID-19 disease. In severe cases, patients suffer from acute respiratory distress syndrome usually associated with an increased level of inflammatory cytokines, called a cytokine storm. It seems that reequilibrating the hyperinflammatory response of the host immune system and regeneration of damaged cells could be the main way to manage the disease. Mesenchymal stem cells (MSCs) have been recently under investigation in this regard, and the achieved clinical outcomes show promising evidence for stem cell-based therapy of COVID-19. MSCs are known for their potential for immunomodulation, defense against virus infection, and tissue regeneration. MSCs are a newly emerged platform for designing vaccines and show promising evidence in this area. In the present study, we provided a thorough research study on the most recent clinical studies based on stem cells in the treatment of COVID-19 while introducing stem cell exclusivities for use as an immune disorder or lung cell therapy and its potential application for protection and vaccination against COVID-19.

## 1. Introduction

New coronavirus (COVID-19) disease has been a pandemic catastrophe since January 2020, when the World Health Organization (WHO) declared COVID-19 as a global health emergency. Severe acute respiratory syndrome coronavirus 2 (SARS-CoV-2) has a 27-32 kb nucleotide genome composed of a single-stranded RNA. The 8 nonstructural and the 4 structural proteins (including membrane (M), nucleocapsid (N), envelope (E), and spike (S)) are encoded by the virus genome [1, 2]. This virus is transmitted very quickly around the world and has affected millions of people so far. Infection with this virus mostly results in either none or mild symptoms (flu-like signs), is self-limited, and needs no preferential treatment. However, 2-6% of the patients demonstrate acute respiratory distress syndrome (ARDS), lung fibrosis, and multiorgan failure that might lead to death [3]. The mortality rate of COVID-19 is reported to vary in different ages and nations [4]. Due to the highly contagious quality of the SARS-CoV-2 virus, it is estimated that many people may die from COVID-19 infection all over the world. Many preventive and treatment medications have been recommended since the diagnosis of this virus, and lots of research are under consideration to provide an approved medication. From a pathophysiological point of view, COVID-19 is usually associated with elevated levels of inflammatory cytokines including interleukin- (IL-) 2, IL-6, IL-7, granulocyte colony-stimulating factor (GSCF), interferon-inducible protein 10 (IP10), monocyte chemotactic protein 1 (MCP1), macrophage inflammatory protein 1A (MIP1A), and TNF-*α* [4, 5]. This condition is called cytokine storm. Besides, COVID-19 in severe cases is associated with ARDS and the damage to alveolar epithelial and endothelial cells. It seems that antiviral, anti-inflammatory, and cell regenerative therapies as well as supportive care during the period of the disease are the main treatments for COVID-19 [6]. A review of recent studies showed that biological interventions are promising for the management of inflammatory responses and tissue treatment of degenerative diseases. Of these, mesenchymal stem cell (MSC) therapy has found a special place in the therapeutic research of COVID-19 [7]. MSC-based treatments have been applied in several autoimmune disorders successfully and are approved to be a safe treatment [8, 9]. It seems that MSCs can exert a profound effect on COVID-19 treatment relying on its immunomodulatory effect and regenerating potential. The present study focuses on stem cell characteristics and introduces a brief review on the most recent stem cell-based clinical studies for the treatment of COVID-19. We also focused on the molecular aspects of MSCs, their unique features to be used as a preventive factor against COVID-19, and vaccine design.

This study represents the results from a thorough survey in several databases of MEDLINE/PubMed, Web of Science, Embase, and Google Scholar regarding the terms “COVID-19” and “stem cell” from 19 October 2020. We extracted clinical trial registries using the WHO International Clinical Trials Registry Platform (ICTRP) and clinicaltrials.gov to provide any stem cell-based intervention for COVID-19 treatment from December 2019 to October 2020. Both clinical trial and experimental studies on the effect of stem cell-based strategies for COVID-19 treatment were included in this study. Besides, the most relevant documents considering “stem cell biology,” “immunomodulatory effects of stem cells,” and “regenerative and anti-apoptotic effects of stem cells” were also included for the interpretation of documented results.

## 2. MSCs for Treatment of Lung Diseases and Immune Disorders

One of the main features of ARDS is the disruption of the alveolar-capillary membrane and apoptosis of alveolar epithelial and endothelial cells. Apoptotic cells attract inflammatory cells and lead to lung tissue regeneration. Based on recent in vivo studies, MSCs can inhibit from apoptosis of the alveolar epithelial cells and endothelial cells due to the secretion of growth factors such as keratinocyte growth factor (KGF), angiopoietin-1 (Ang1), and hepatocyte growth factor (HGF) and reduction of tumor necrosis factor-*α* (TNF-*α*) [10–13]. Due to the decreased pulmonary permeability and increased alveolar edema and intrapulmonary shunting, ARDS is commonly associated with hypoxemia [14]. MSCs can be stimulated in response to the hypoxic stress from the lung tissue [15]. It has been demonstrated that in hypoxic conditions, the secretome of adipose-derived MSCs is enriched with angiogenic factors and improves angiogenesis in an in vivo mouse model [16]. Elevated secretion of Ang1 and KGF from bone marrow-derived MSCs was recently reported to attenuate inflammation and inhibit alveolar epithelial cells from apoptosis [11]. It seems that MSCs are promising candidates for airway treatment considering their angiogenic properties and their ability to reduce alveolar cell apoptosis. According to previous studies, MSCs have been tested in animal models and clinical trials for the treatment of lung diseases and immune disorders. It has been demonstrated that MSCs can exert an immunomodulatory effect on mice with LPS-mediated lung injury through stanniocalcin-2 protein [17]. MSC therapy is now an approved medication for the treatment of several immune diseases including multiple sclerosis and Crohn's disease [8]. Moreover, promising results are available for MSCs application in animal models for the treatment of lung disease (such as idiopathic pulmonary fibrosis, chronic obstructive bronchiolitis, and bronchopulmonary dysplasia) [18–20].

### 2.1. Clinical Application of MSCs for COVID-19 Treatment

According to recent studies, MSC therapy seems to be an innovative treatment for COVID-19, as it involves lung tissue and is associated with inflammatory responses. Stem cells are considered a choice in the treatment of diseases with high morbidity rates, based on FDA regulations [21]. By the time of preparing this paper, 88 stem cell-based clinical trials were registered in clinicaltrials.gov and ICTRP portal for treatment of COVID-19. Different sources have been used for extraction and expansion of MSCs in these trials according to the details provided by clinicaltrials.gov and ICTRP portal. Most of these clinical trials were conducted in China, and the outcomes showed the safety of stem cell-based treatments to cure severe cases of COVID-19. Stem cell-based therapies for COVID-19 are mainly administered as an intravenous injection (IV) of MSCs suspension; however, the application of extracellular vesicles secreted by stem cells has also been reported.

### 2.2. IV of MSCs

The therapeutic effect of MSCs in the treatment of COVID-19 has been evaluated in several clinical trials in recent months considering its safety and efficacy. A brief review of registered clinical trials in clinicaltrials.gov and ICTRP portal is presented in Table 1. As shown in Table 1, umbilical cord-derived MSCs are reported to be the most used stem cells, and the main route of administration is IV. MSCs used for these trials were mostly extracted from autologous or allogenic tissue sources and expanded in clinical-grade clean rooms. After the assessment of specific markers (positive for surface markers CD 73, CD 90, and CD105; negative for surface markers CD11b, CD14, CD19, CD34, CD45, CD79a, and HLA-DR) and confirmation of noncontamination, the cells were usually suspended in a standard buffer (mostly normal saline) for infusion into the patients. The dose of suspended cells/ml or cells/kg, the number of injections, and the time of intervals between injections vary in different studies (see Table 1). Although many of the registered studies are still recruiting, the the primary clinical outcomes clinical outcomes suggest that MSC therapy is promising for COVID-19 treatment, particularly in severe cases (see below).

### 2.3. Conditioned Medium and Extracellular Vesicles Derived from Stem Cells

Extracellular vesicles (EVs) consist of exosomes and microvesicles with a diameter of 0.03 to 1 *μ*m, secreted by all cell types. EV contains macromolecules secreted by the cell source and is the main factor in intercellular communication through paracrine secretion [22, 23]. The effect of MSC-EV on the treatment of viral and bacterial infections of the lung has recently been tested in several animal and human models. *Escherichia coli*-mediated severe pneumonia in ex vivo models of human lung injury [24], influenza-induced lung injury in pig models [25], and bacterial sepsis in murine models [26, 27] was successfully cured by using MSC-EV or MSC-conditioned medium. In a recent study, it has been shown that conditioned medium produced by adipose-derived MSCs (ad-MSCs) could significantly reduce lung injury in murine models. The increased alveolar fluid clearance, prevention of lung from fibrosis, and improving the lung function were of the reported effect of MSC-EV on lung injury [28–30]. It is suggested that the macromolecules present in the secretome of MSCs are primarily responsible for the immunomodulatory and regenerative effects of MSCs. MSC-EVs have been applied for the management of the COVID-19 pandemic and registered in five clinical trials. The route of administration is EV infusion or EV inhalation. Recently, a trial was performed on 24 COVID-19 patients utilizing EV infusion for the treatment of COVID-19 pneumonia [31]. After a follow-up period of 14 days, the safety of MSC-EVs was approved, and no severe adverse effect was documented. The clinical signs were improved by approximately 80% of patients. Although this study included a small population and the efficacy of EV therapy could not be concluded just from this report, the potential therapeutic effects of EVs on COVID-19-induced lung injuries should be taken into account. Regarding the safety challenges of cell therapy, EVs seem to be much safer than their parent cells [32, 33]. EVs can readily diffuse through tissues and cell barriers regarding their nanodiameter [33]. There is evidence to suggest that MSC-mediated immunomodulatory and regenerative properties are due to the vesicles originating from them.

### 2.4. General and Unique Properties of MSCs

Mesenchymal stromal stem cells are a heterogeneous population of cells mostly derived from two natural stem cell sources: embryonic stem cells (ESCs) and adult stem cells [34]. Adult stem cell sources such as the Wharton jelly, umbilical cord, placenta, bone marrow, adipose tissue, and dental pulp have more advantages than ESCs, considering ethical problems and risk of tumorigenesis [34, 35]. Umbilical cord is of the most common, safe, and reliable sources of stem cell. The similarity of their gene expression profile to embryonic stem cells and no sign of tumorigenicity is an advantage of umbilical cord MSCs compared to ESCs [36].

MSCs are multipotent cells, known for their unique potential to regenerate damaged cells and their immunomodulatory properties [4, 37]. They can secrete a broad range of cytokines and growth factors as soluble factors or extracellular vesicles in their condition medium. Previous studies have shown that MSCs are promising for the treatment of inflammatory or autoimmune diseases [38]. It seems that the palliative effect of MSCs on inflammatory diseases is due to their secretion profile of immunomodulatory proteins [39], although this profile can vary according to cell source and cell passage number [5, 40]. Stem cells have other unique characteristics that make them a hopeful candidate for COVID-19 treatment. Low expression of major histocompatibility complex (MHC) makes stem cells nonimmunogenic in host patients [41]. MSCs do not express ACE2 and TMPRSS2 receptors (the virus entry gate into the host cell), a beneficial point for COVID-19 treatment [42]. The lack of these receptors on MSCs suggests that the COVID-19 virus is not able enter MSCs and infect them. MSCs can communicate with host immune cells both by cell-cell-mediated communication and by secreting soluble factors and microvesicles [43]. Recent studies have shown that the therapeutic effect of MSCs in lung injury can be due to the secretion of factors such as nitric oxide (NO), transforming growth factor-*β* (TGF-*β*), prostaglandin E2 (PGE2), indoleamine2,3-dioxygenase (IDO), KGF, and Ang1 [27, 44]. PGE2 induces the reprogramming of alveolar macrophages from the M1 phenotype to the alternative M2 phenotype that can release IL-10 and promote the resolution of inflammation [45, 46]. PGE2 can also inhibit T cell-dependent inflammation. NO, IDO, and TGF-*β* are the other factors that can suppress T cell-dependent inflammation [47–49]. KGF as a cytoprotective factor and Ang1 as an antipermeability agent play a pivotal role in cell regeneration of damaged lung tissue [27].

Stem cells can also downregulate the production of interferon-*γ* (IFN-*γ*), TNF-*α*, and IL-17 and upregulate the production of IL-10 that results in modulation of host immune response. MSCs can suppress CD8^+^ cells and natural killer (NK) cells as well, which leads to the inhibition of T cell-mediated damage to the lung parenchyma [27, 44]. MSCs release fibrinolytic factors that might be useful for the elimination of pulmonary fibrosis [50]. MSCs can regulate the immune system by activating or suppressing the immune system (according to the around stimuli) [4, 37]. It should be noted that the effect of modulating the immune system of mesenchymal stem cells is not long-lasting and disappears between six months to one year; however, due to the nonchronic nature of COVID-19, even the effect of temporarily modulating the MSC on the immune system can significantly help COVID-19 patients. [51].

### 2.5. The Most Recent Reports Available on COVID-19 Cell Therapies

In this section, the most recent reports on the completed trials of COVID-19 cell therapies are provided. The primary results are indicative of the safety and efficacy of MSCs therapy of COVID-19.

**ChiCTR2000029990**: Leng et al. were the first to report the successfulness of a pilot study on COVID-19 MSCs therapy. They reported that intravenous injection of MSCs into 7 patients infected with COVID-19 revealed encouraging outcomes and improved the clinical signs of patients. Lymphopenia, fever, shortness of breath, respiratory rate, and pneumonia infiltration were significantly recovered in these patients, and no adverse effect was reported. CXCR_3_^+^ CD_4_^+^ T cells, CXCR_3_^+^ CD_8_^+^ T cells, and CXCR_3_^+^ CD_4_^+^ NK cells disappeared after 3-6 days of MSC injection. The decreased amount of TNF-*α* and increased level of IL-10 were also observed in the treated patients [42].

**NCT04252118**: In this clinical study, intravenous injection of UC-MSCs (3 × 10^7^ cells per infusion) was used for 18 enrolled COVID-19 patients, and clinical outcomes were recorded on days 0, 3, and 6. No serious infusion-associated complication was observed, and all patients were recovered. The results of this study showed that UC-MSC IV was safe and well tolerated in patients with moderate and severe COVID-19 [52].

**NCT04269525**: The safety and feasibility of UC-MSC transplantation in COVID-19 were evaluated in this trial on sixteen patients with severe and critically severe conditions. No allergic reaction and adverse events were observed until 28 days after transplantation. The mortality rate decreased significantly, and oxygenation index, cytokine levels, radiological images, and lymphocyte count were improved. This study also confirmed the safety and feasibility of IV of UC-MSCs in severe cases of COVID-19 pneumonia [53].

**NCT04355728**: This study was a phase 1/2a clinical trial which was held on 24 subjects (12 per group) in Miami, Florida, USA. Participants of each group were intravenously injected with 100 × 10^6^ UC-MSC, or vehicle, at days 0 and 3. Twenty-eight days follow-up showed that mortality rate and time of recovery were decreased significantly in the UC-MSC group compared to the control one. Besides, no serious adverse events were recorded [54].

**NCT04428801**: Ad-MSCs were applied in this study as one of the other MSC sources for COVID-19 stem cell therapy. The safety and prophylactic efficacy of Ad-MSCs were evaluated in a Phase II study held by Celltex (TX, USA; http://www.celltexbank.com) [55]. In this study, 200 patients were enrolled from which 100 patients were intravenously injected with autologous Ad-MSCs and 100 with placebo treatments. This cell product received FDA approval as a prophylactic against COVID-19, and the study is still recruiting.

**ChiCTR2000030261**: This pilot study focused on MSC-derived exosomes for the treatment of seven patients with COVID-19 pneumonia. Exosomes secreted by MSCs were purified using multiple ultrafiltration and administered as a respiratory spray. The results of this study showed that aerosol inhalation of MSC-EV was not associated with any allergic reaction; besides, it improved the pulmonary lesions in COVID-19 patients. This study confirmed the safety and efficiency of nebulization of MSC-EV. The simple method used for the production of this substrate was also a beneficial point for this strategy [56].

According to the above-mentioned reports, MSCs and MSC-EVs are interesting and hopeful candidates for COVID-19 treatments. Although there are many other studies recruiting, it seems that MSC therapy is suitable for severe and critically severe COVID-19 patients.

### 2.6. The Molecular Aspects of MSCs against COVID-19: How Stem Cells Can Be Preventive against COVID-19?

The cellular immune response varies in differentiated and stem cells in certain aspects. IFN production is the most common response of differentiated cells to viral infection [57]. When viruses enter the host cell, stimulate IFN production, and subsequently trigger the transcription of a wide range of interferon-stimulating genes (ISGs) in various levels based on the cell type [57], this is the main pathway for the induction of viral defense in differentiated cells and is called the innate immune response. SARS-CoV-2 virus appears to exert its effect by inhibiting IFN production in human cells [57]. Impairment of innate immune response extends SARS-CoV-2 virus incubation time (2-14 days) and allows virus replication to high titration. Providing an immune-supportive agent seems to help inhibit from COVID-19 infection, particularly at the early stages of the disease. Stem cells have distinct characteristics that make them resistant to viral infections [58]. It has been shown that stem cells express ISGs continuously even in the absence of viral infection [59]. The IFN-independent ISGs expression is the basis of stem cells' intrinsic viral resistance [59]. Many studies have confirmed the resistance of pluri/multipotent cells against different pathogenic infections [60, 61]. In contrast, differentiated cells are readily infected with viruses [62]. One of the important aspects of stem cells is that they do not generate type-I IFN in response to pathogens and are refractory to extrinsic IFNs [63, 64]. So, it is suggested that canonical IFN signaling is not the main pathway for viral resistance in stem cells [58]. In other words, stem cells utilize an IFN-independent arrangement which is based on ISG protein expression to defend against the virus. This characteristic of stem cells can be considered a feature that is effective against COVID-19. Considering the property of SARS-CoV-2 virus for IFN shut down in infected cells, it can be suggested that stem cells can be utilized as a supportive source of cells for enhancing the innate immune response to this virus specifically at the early phase of infection. Recently, it has been shown that ISG expression can stop virus replication at the initial stages of the virus life cycle [65]. In a study by Wu et al., ISGs expression was analyzed in 3 germ layer-specific stem cells [58]. According to their report, mesenchymal stem cells express higher numbers of ISGs compared with pancreatic stem cells and neural stem cells [58]. Although ISG expression pattern is distinct to each type of stem cell, the family gene of IFITM was expressed at high levels in all 3 cell types [58]. IFITM genes are a component of ISGs that inhibit virus entrance to the cell and replication [66]. IFITM overexpression on the stem cell membrane is a unique property of stem cells to warrant their security against viruses.

Another macromolecule that has recently come to the attention of researchers is leukemia inhibitory factor (LIF). LIF is a stem cell growth factor that can counteract cytokine storms in the lungs during viral pneumonia [67]. This vital protein controls inflammation in the lungs and at the same time repairs lateral damage to small blood capillaries and alveoli. MSCs are of the well-known sources of soluble LIF [68]. Recently, LIF has been introduced as an attractive treatment option for multiple sclerosis and neurodegenerative diseases associated with inflammation since LIF has been shown to repair damaged neurons in the brain and spinal cord of the central nervous system [69]. One of the important clinical features of LIF is that it can directly inhibit IL-6 activity, thereby preventing cytokine storms [70]. Targeting the IL-6 pathway is an interesting clinical approach to reduce COVID-19 mortality. The use of tocilizumab human monoclonal antibody to bind and block IL-6 receptors is one of these studies [71]. LIF is suggested to be used not only as a therapeutic option but also as a protective agent against the pathogenesis of COVID-19 progression. It has been shown that the LIF/IL-6 axis can correct the inflammatory response of the host and not only protects the lungs against chronic diseases but also speeds up the regeneration of other organs after COVID-19 is eliminated, although there exists a challenge associated with the burden cost of providing sufficient LIF for treatment. Recently, using nanotechnology synthetic stem cells, a product called “LIFNano” has been invented that has shown a thousand times more power than soluble LIF [72]. In an animal study, treatment with LIFNano was performed on a preclinical model of multiple sclerosis (MS), and the result was cured paralysis within 4 days [72]. This short time frame for clinical outcomes is consistent with reporting the beneficial effects of MSC treatment on COVID-19 pneumonia. LIFNano is a newly emerging alternative to cell-based therapy that can regenerate damaged tissues and suppress cytokine storms in pneumonia by providing nanoparticles of active LIF that are a thousand times more potent than soluble LIF. LIF seems to be able to reduce the severity of the disease and thus reduce the pressure on global health systems. The suggested routes of administration are direct delivery of LIF to the respiratory system (nasal inhalation) or intravenous injection [70, 73, 74].

Besides the molecular aspects mentioned above, there is a huge bulk of studies which indicate the protective ability of MSC against the inflammation-induced damage of pulmonary endothelial cells and alveolar epithelial cell. The secretome of MSCs is composed of several cell-protective bioagents each of which is of a pivotal role in the protection and restoration of impaired alveolar cells [13, 75]. Ang1, PGE2, HGF, and KGF are the protective factors secreted by MSCs that inhibit alveolar cells from inflammation and oxidative damages [10, 76, 77]. Studies in animal models of ARDS have shown that MSCs can upregulate the level of metalloproteinase- (MMP-) 8 and downregulate the expression of tissue inhibitor of metalloproteinase- (TIMP-) 1, IL-1*β*, and TGF-*β*1 to attenuate the lung fibrosis [78, 79].

Finally, we want to hypothesize that more functional stem cells in the body might be able to prevent COVID-19 infection or decrease the symptoms by increasing the ability to repair, protecting the lung cells, and immunomodulatory properties. An evidence-based observation is the lower rate of contagion, milder symptoms, and a lower rate of mortality of COVID-19 in younger patients [80, 81]. Reports are indicating that although people of all ages can be infected by this virus, the disease progresses faster and shows more severe symptoms in the elderly than in the young [82]. This might be directly related to the aging-associated dysfunction of stem cells in the elderly [83]. Perhaps the proliferating stem cells in young people are playing a defensive role against COVID-19 infection. However, the observations just provide basic evidence for the possible effect of stem cells in prevention against COVID-19, and there is still a long way to apply this modality in humans.

### 2.7. Can Stem Cells Be Used for COVID-19 Vaccination?

Vaccination seems to be the most effective means of prevention against COVID-19 contagion. Recently, many studies have been conducted to develop the COVID-19 vaccine, and many options have been proposed, including DNA- and RNA-based engineered vaccines, but there are still limitations and warning points about the vaccines, including efficacy, tolerability, safety, and immune adaptability [84]. New approaches to vaccine development have been introduced recently, including cellular vaccine strategies which have been mostly used against cancer [85, 86]. Cellular vaccines benefit from the direct transfer of transfected host cells for in vivo production of vaccine antigens. Mesenchymal stem cells are a newfound platform for designing genetically engineered cellular vaccines that hold the promise to produce efficient and safe vehicles for enhancing the host immune response [87]. Using human-engineered mesenchymal stem cells (hu-MSCs) to produce the SARS-CoV-2 N-protein antigen is one of the promising strategies that has been reported in a recent paper of Chinese researchers as a candidate for producing the COVID-19 vaccine [88]. They showed that mesenchymal stem cells engineered to produce COVID-19 proteins could provide a new bench for effective vaccine development. In this proof of concept study, transfected allogeneic mesenchymal stem cells (carrying plasmids for SARS-CoV-2 N-protein) were applied for mice injection. Twenty days after intramuscularly or subcutaneously vaccination, blood samples from the mice were tested for detection of antibodies against N-protein using enzyme-linked immunosorbent assay (ELISA). The results of this study demonstrated that one dose of vaccination led to positive antibody production in the serum of injected mice. Moreover, the MSCs carrying the transfected gene are cleared from the bloodstream by the immune system 20 days after injection.

To be more successful in using engineered mesenchymal cells as vaccines, MSCs must be as invisible to the recipient's immune system as possible [89]. In a recent study, human mesenchymal stem cells were genetically engineered using the MSCVneo retrovirus so that HLA-1 expression was downregulated [90]. Subsequent studies showed that the injection of these stem cells was associated with a decrease in the proliferation of mononuclear cells in animal models and human subjects [90]. Future directions in this field may include strategies to modify the transfected proteins in the stem cells by posttranslational processes such as acetylation and glycosylation. In this way, the engineered antigen becomes more like viral proteins in vivo, can simulate the host immune response much better, and increase the success rate of immune protection.

### 2.8. Challenges to Use MSCs

Although clinical observations indicate the high potential of MSCs in the treatment of fatal diseases such as COVID-19, there are still several issues associated with the administration of MSCs. The heterogeneous source of MSCs, methods of preparation, number of MSCs, and the age of the cell population can certainly lead to various functional differences. This variation might be in the potential of cytokine release, cytokine content, and the therapeutic capacity [27]. Even the variation in size, shape, and passage number of MSCs or MSC-EVs may also lead to different therapeutic effects of this biological substrate. In a study by Kowal et al., it has been shown that different sizes of EVs derived from dendritic cells can exert different effects on T cells [91]. The cost and speed of sample preparation are also issues that should be taken into consideration. Although MSC-EVs seem to be much cheaper and easier to large-scale production compared to their cells of origin. Another requirement for MSC therapeutic application is a well-established protocol that fulfills the criteria of Good Manufacturing Practice (GMP) for the production of clinical-grade MSCs [92]. Moreover, standard methods are required for producing MSCs or MSC-EVs in a high degree of purity appropriate for use at the industrial level. The produced MSCs for therapeutic applications should be evaluated considering the criteria recommended by the international society for cell therapy (ISCT). The presence of certain surface markers, analysis of protein content, secretome, MSC-EVs, and microRNAs should be approved by using standard assays before any therapeutic applications [93, 94]. It seems that a reliable cell bank for production, quality control, and storage of standard MSC vials might compensate for these challenges in an emergency condition.

Another main challenge of using MSCs therapy for COVID-19 patients is that COVID-19-infected patients are at the risk of hypercoagulation [95]. MSCs have been shown to possess procoagulation properties. Recently, Moll et al. have nicely provided the safety hazards and applicable protocols for the preparation of MSCs to overcome this problem [96]. It is strongly recommended that well-characterized cell products, checked for rare expression of tissue factor (TF/CD142) on their surface, be used for clinical application. Also, consideration of nonintravascular routes of MSC or MSC-EV administration is encouraged to reduce the risk and improve the safety and efficacy of COVID-19 cell therapy.

## 3. Conclusion

According to recent reports, MSC-based treatments seem to be an innovative biological intervention for the treatment of COVID-19. Considering the exclusivity of MSCs to defend against viruses, immunomodulatory properties, and their potential for tissue regeneration, MSC-based treatments deserve more attention from researchers and seem to be applied more to treat COVID-19. However, there are several challenges associated with cell-based therapies. Perhaps, using a standard and creative approach to stem cell utilization can solve previous challenges.

## Figures and Tables

**Table 1:** A brief review of recent clinical trials using stem cell-based therapy for treatment of COVID-19.

Stem cell source: umbilical cord-derived stem cells (UC, WJ, placental–MSCs)			
*N* (age)	Route of administration	S	Clinical trial ID
20 (18-70)	3× IV (3 × 10^7^ cells/infusion at d 0, 3, 6)	R	NCT04252118 (China)
30 (≥18)	3× IV (details not specified)	R	ChiCTR2000030866 (China)
20 (16-75)	3× IV (2 doses: (10^6^ cells/kg in 1.25 ml) and (2 × 10^6^ cells/kg in 2.5 ml))	R	ChiCTR2000030835 (China)
16 (18-80)	4× IV (3.3 × 10^7^/50 ml/bag, 3 bags each time at days 1, 3, 5, and 7)	R	NCT04269525 (China)
9 (18-75)	IV (details not specified)	R	ChiCTR2000030300 (China)
48 (18-65)	4× IV (5 × 10^6^/kg at days 1, 3, 5, and 7)	NR	NCT04273646 (China)
60 (18-70)	(details not specified)	NR	ChiCTR2000030173 (China)
60 (16-75)	IV (details not specified)	NR	ChiCTR2000030138 (China)
16 (18-75)	IV (comparison of different doses, details not specified)	R	ChiCTR2000030116 (China)
60 (≥18)	IV (details not specified)	NR	ChiCTR2000029816 (China)
[17–74]	4× IV (0.5 × 10^6^/kg in 100 ml)	W	NCT04293692 (China)
10 (18-95)	3× IV (0.5 − 1 × 10^6^ at days 1, 3, and 6)	NR	IRCT20140528017891N8 (Iran)
40 (≥18)	1× IV (details not specified)	C	NCT04573270 (USA)
70 (≥18)	IV (at day 1, second infusion at day 7 according to physician discretion)	R	NCT04565665 (USA)
30 (18-70)	IV (1 × 10^6^/kg)	NR	IRCT20200426047206N2 (Iran)
20 (18-70)	3× IV (1 × 10^6^/kg at days 1, 3, and 6)	R	IRCT20160809029275N1 (Iran)
30 (18-79)	IV (1 × 10^6^ cells/kg)	NR	NCT04429763
30 (18-75)	IV (1 × 10^6^ cell/kg in 100 ml of NS, second infusion at d 7 according to physician discretion)	R	NCT04339660 (China)
20 (30-70)	3× IV (5 × 10^5^ cell/kg at d 1, 3, 5)	R	NCT04437823 (Pakistan)
21 (≥18)	2× IV (100 × 10^6^ cell/kg at days 0 and 3)	NR	NCT04490486 (USA)
60 (18-70)	4× IV (10^6^ cell/kg/time every 4 days)	NR	NCT04371601 (China)
60 (≥18)	1 or 2× IV by 48 h interval (10^6^ cell/dose in NS)	R	NCT04494386 (USA)
40 (18-95)	1× IV (1 × 10^6^/kg in 100 ml of NS)	R	NCT04457609 (Indonesia)
40 (18-80)	IV (1 × 10^6^ cell/kg in 40 ml)	R	ChiCTR2000030088 (China)
5 (≥18)	3× IV (10^6^ cell/kg in 25 ml every 3 days)	R	NCT04313322 (Jordan)
40 (≥18)	3× IV (1 × 10^6^ cell/kg in a 150 ml, at days 1, 3, and 5)	R	NCT04333368 (France)
20 (18-65)	IV (2 doses: (10^6^ cells/kg in 1.25 ml) and (2 × 10^6^ cells/kg in 2.5 ml) at days 1 and 4)	NR	CTRI/2020/08/027043 (India)
20 (18-65)	1× IV (3 doses: (100 × 10^6^ cells (±10%) at days 0, 2, and 4)	R	IRCT20200413047063N1 (Iran)
6 (no limit)	3× IV (1 × 10^6^ cells/kg, once every 3-4 days during a 14-day period)	R	IRCT20200418047121N2 (Iran)
90 (18-85)	IV (0.5 − 2 × 10^6^ cell/kg, at days 1, 3, and 6)	R	IRCT20200421047150N1 (Iran)
40 (18-80)	IV (50 × 10^6^)	NR	NCT04390152 (Colombia)
9 (≥18)	1× IV (1 × 10^8^ cells)	NR	NCT04456361 (Mexico)
30 (18-75)	1× IV (1 × 108 cells/kg at days 1 and 3)	R	NCT04390139 (Spain)
24 (≥18)	IV (100 × 10^6^ cells)	NR	NCT04355728 (USA)
Stem cell source: BM-MSCs			
*N* (age)	Route of administration	S	Clinical trial ID
20 (18-75)	IV (1 × 10^6^/kg at day 1)	NR	NCT04346368 (China)
20 (≥10)	IV (2 × 10^6^ cells/kg at day 1, second infusion at day 7 according to physician discretion)	R	NCT04444271 (Pakistan)
40 (≥18)	IV (details not specified)	NR	NCT04377334 (Germany)
45 (18-80)	IV (details not specified)	R	NCT04397796 (USA)
9 (18-65)	1× IV (2 doses: (1 × 10^6^ cells/kg) and (1 × 10^6^ cells/kg))	R	NCT04447833 (Sweden)
Stem cell source: Ad-MSCs			
*N* (age)	Route of administration	S	Clinical trial ID
26 (18-80)	IV (80 × 10^6^ cells)	R	NCT04366323 (Spain)
100 (≥18)	IV (2 serial doses of 1.5 × 10^6^ cells/kg)	NR	NCT04348461
20 (18-90)	IV (5 × 10^5^ cells)	NR	NCT04352803
10 (19-80)	(details not specified)	NR	NCT04527224
6 (≥20)	4× IV (1 × 10^8^ cells, once a week)	NR	JPRN-JapicCTI-205416 (Japan)
0 (18-80)	IV (100 × 10^6^ cells in 100 ml NS)	NR	NCT04341610 (Denmark)
56 (NL)	5 × IV (details not specified)	E	NCT04349631 (USA)
100 (NL)	5× IV (3 doses: (50 × 10^6^ cells), (100 × 10^6^ cells), and (200 × 10^6^ cells) at weeks 0, 2, 6, 10, and 14)	E	NCT04348435 (United States)
20 (18-80)	(details not specified)	NR	NCT04486001 (USA)
200 (≥18)	IV (200 × 10^6^ cells, every three days)	NR	NCT04428801
100 (NL)	4× IV (100 × 10^6^ cells at days 0, 3, 7, and 10)	NR	NCT04362189 (USA)
Stem cell source: Dp-MSCs			
*N* (age)	Route of administration	S	Clinical trial ID
24 (18-75)	3× IV (10^6^ cell/kg in 50 ml NS)	NR	NCT04302519 (China)
20 (18-65)	3× IV (3 × 10^7^ cells in 30 ml at days 1, 4, and 7)	R	NCT04336254(China)
20 (18-65)	IV (details not specified)	NR	ChiCTR2000031319 (China)
10 (18-95)	1× IV (40 × 10^6^ cells)	R	IRCT20140911019125N6 (Iran)
Stem cell source: not specified			
*N* (age)	Route of administration	S	Clinical trial ID
120 (18–95)	IV (1 × 10^6^ cells/kg in 100 ml of NS)	R	ChiCTR2000029990 (China)
100 (18-75)	3× IV (4 × 10^7^ cells/time at days 0, 3, and 6)	C	NCT04288102 (China)
120 (18-95)	(details not specified)	R	ChiCTR2000029990 (China)
20 (18-70)	4× IV (details not specified)	R	ChiCTR2000030020 (China)
32 (18-100)	IV (details not specified)	NR	ChiCTR2000030224 (China)
90 (≥18)	IV (3 or 4 cycles of 2 × 10^7^ cells at days 1, 3, 5, and 7)	NR	NCT04315987 (Brazil)
9 (18-70)	IV (3 doses: 3 × 10^6^, 5 × 10^6^, and 10 × 10^6^ cells/kg)	R	NCT04331613 (China)
200 (18-80)	3× IV (details not specified)	R	ChiCTR2000031430 (China)
36 (18-90)	IV (details not specified)	R	ChiCTR2000031494 (China)
5 (18-70)	3× IV (70 × 10^6^ cells at days 0, 3, and 6)	NR	IRCT20200325046860N2 (Iran)
30 (40-60)	IV (3 × 10^6^ cells/kg at days 0, 3, and 6)	R	NCT04392778 (Turkey)
10 (≥18)	1× IV (1 × 10^6^ cells/kg)	R	NCT04416139 (Mexico)
6 (18-70)	3× IV (200 × 10^6^ (±10%) cells in 50 ml NS at days 0, 2, and 4)	NR	IRCT20200217046526N1 (Iran)
300 (≥18)	2 × 10^6^ cells/kg, second infusion at 4 days following the first infusion (±1 day)	R	NCT04371393 (USA)
400 (18-89)	IV (details not specified)	R	NCT04367077 (USA)
24 (≥18)	IV (2 × 10^6^ cells/kg at days 1 and 3)	R	NCT04537351 (Australia)
9 (≥19)	IV (2 doses: (5 × 10^7^ cells) and (1 × 10^8^ cells))	NR	NCT04535856 (Indonesia)
MSC-EVs			
*N* (age)	Route of administration	S	Clinical trial ID
24 (18-75)	5× aerosol inhalation (2 × 10^8^ EV/3 ml at days 1, 2, 3, 4, and 5)	C	NCT04276987 (China)
26 (18-65)	Aerosol inhalation (details not specified)	NR	ChiCTR2000030261 (China)
64 (≥70)	IV (0.2 mg/kg in 15 ml at days 1 and 3)	R	ISRCTN33578935 (Germany)
MSCs + MSC-EVs			
*N* (age)	Route of administration	S	Clinical trial ID
90 (18-70)	UC-MSCs and exosomes IV (5 × 10^7^ cells/time, 1 time/week, 2 times/course, a total of 2 courses; exosomes: 180 mg/time, 1 time/day, 7 days/course, 2 courses in total)	NR	ChiCTR2000030484 (China)
60 (18-65)	MSCs + EVs 2× IV (3 doses: 100 × 10^6^ (±10%) cells at days 0 and 2) 2 × IV (MSC-EVs at d 4, 6)	R	NCT04366063 (IRCT20200217046526N2) (Iran)
Stem cell-based combination therapies			
*N* (age)	Route of administration	S	Clinical trial ID
70 (18-75)	MSCs + ruxolitinib (details not specified)	R	ChiCTR2000029580 (China)
30 (≥18)	UCB-MNC CM (details not specified)	NR	ChiCTR2000029569 (China)
75 (≥16)	UC-MSCs enriched with CD362 IV (maximum tolerated dose from the phase 1 trial)	R	NCT03042143 (UK)
20 (4-80)	NK + UC-MSCs (details not specified)	NR	ChiCTR2000030944 (China)
60 (≥18)	UCB-NK + UCB-MSC 3 doses: 5× IV (5 × 10^9^ + 5 × 10^9^ every 2 days) and 3× IV (3 × 10^9^ NK + 3 × 10^9^ MSC every 2 days), and 1 IV (3 × 10^9^ NK + 3 × 10^9^ MSC in a week)	NR	ChiCTR2000029817 (China)
600 (18-90)	Convalescent plasma, mesenchymal stem cell therapy, remdesivir, and Tocilizumab, alone or in combination IV (2 doses: [100 × 10^6^ cells]and [200 × 10^6^ cells] at d 1, 4)	NR	NCT04492501 (Pakistan)
86 (≥18)	Human placental hematopoietic stem cell-derived NK cells (CYNK-001) IV (at days 1, 4, 7; details not specified)	R	NCT04365101 (USA)
146 (≥18)	Autologous nonhematopoietic peripheral blood stem cells (NHPBSC) (details not specified)	NR	NCT04473170 (United Arab Emirates)
30 (18-75)	Cryopreserved allogeneic P_MMSCs and UC-MMSCs 3× IV (1 × 10^6^ cells/kg/time, once every 3 days at days 1, 4, and 7)	R	NCT04461925 (Ukraine)
40 (18-70)	Allogenic pooled olfactory mucosa-derived MSCs (details not specified)	E	NCT04382547 (Belarus)
63 (1-99)	Hu menstrual blood SC + artificial liver IV (details not specified)	R	ChiCTR2000029606 (China)
20 (18-80)	ESC-derived M cells (CAStem) 2× IV (3 × 10^6^ cells/kg. The interval between each infusion was 1 week (±2 days). Second infusion at day 7 according to physician discretion.)	R	ChiCTR2000031139 (China)

*N*: number of patients, S: status, R: recruiting, NR: not recruiting, NS; normal saline, IV: intravenous injection, NS: normal saline, MSCs: mesenchymal stem cells, UC: umbilical cord, WJ: Wharton jelly, BM: bone marrow, Ad: adipose, Dp: dental pulp, NK: natural killer cell, MSC-EVs: MSC-derived extracellular vesicles, UCB: umbilical cord blood, MNC: mononuclear cells, CM: conditioned medium.

## Abbreviation

Ang1: Angiopoietin-1
ARDS: Acute respiratory distress syndrome
ESCs: Embryonic stem cells
EVs: Extracellular vesicles
GMP: Good manufacturing practice
GSCF: Granulocyte colony-stimulating factor
ICTRP: International Clinical Trials Registry Platform
IDO: Indoleamine2,3-dioxygenase
IFN: Interferon
IL: Interleukin
IP10: Interferon-inducible protein 10
ISCT: International Society for Cell Therapy
ISGs: Interferon-stimulating genes
IV: Intravenous injection
KGF: Keratinocyte growth factor
MCP1: Monocyte chemotactic protein 1
MHC: Major histocompatibility complex
MIP1A: Macrophage inflammatory protein 1A
MSCs: Mesenchymal stem cells
NK cell: Natural killer cell
NO: Nitric oxide
PGE2: Prostaglandin E2
SARS-CoV-2: Severe acute respiratory syndrome coronavirus 2
TGF-*β*: Transforming growth factor-*β*
TNF-*α*: Tumor necrosis factor-*α*
WHO: World Health Organization.
